# Characterizations of Electrospun PVDF-Based Mixed Matrix Membranes with Nanomaterial Additives

**DOI:** 10.3390/nano15151151

**Published:** 2025-07-25

**Authors:** Haya Taleb, Venkatesh Gopal, Sofian Kanan, Raed Hashaikeh, Nidal Hilal, Naif Darwish

**Affiliations:** 1 Department of Chemical and Biological Engineering, College of Engineering, American University of Sharjah, Sharjah P.O. Box 26666, United Arab Emirates; g00098674@aus.edu; 2 Department of Biology, Chemistry & Environmental Sciences, College of Arts and Sciences, American University of Sharjah, Sharjah P.O. Box 26666, United Arab Emirates; vgopal@aus.edu (V.G.); skanan@aus.edu (S.K.); 3 NYUAD Water Research Centre, New York University, Abu Dhabi Campus, Abu Dhabi P.O. Box 129188, United Arab Emirates; rh143@nyu.edu (R.H.); nhh2@nyu.edu (N.H.)

**Keywords:** PVDF, electrospinning, nano-additives, metal–organic frameworks

## Abstract

Water scarcity poses a formidable challenge around the world, especially in arid regions where limited availability of freshwater resources threatens both human well-being and ecosystem sustainability. Membrane-based desalination technologies offer a viable solution to address this issue by providing access to clean water. This work ultimately aims to develop a novel permselective polymeric membrane material to be employed in an electrochemical desalination system. This part of the study addresses the optimization, preparation, and characterization of a polyvinylidene difluoride (PVDF) polymeric membrane using the electrospinning technique. The membranes produced in this work were fabricated under specific operational, environmental, and material parameters. Five different additives and nano-additives, i.e., graphene oxide (GO), carbon nanotubes (CNTs), zinc oxide (ZnO), activated carbon (AC), and a zeolitic imidazolate metal–organic framework (ZIF-8), were used to modify the functionality and selectivity of the prepared PVDF membranes. Each membrane was synthesized at two different levels of additive composition, i.e., 0.18 wt.% and 0.45 wt.% of the entire PVDF polymeric solution. The physiochemical properties of the prepared membranes were characterized by Fourier transform infrared spectroscopy (FTIR), scanning electron microscopy (SEM), zeta potential, contact angle, conductivity, porosity, and pore size distribution. Based on findings of this study, PVDF/GO membrane exhibited superior results, with an electrical conductivity of 5.611 mS/cm, an average pore size of 2.086 µm, and a surface charge of −38.33 mV.

## 1. Introduction

Water scarcity is a pressing global problem, especially in regions where limited freshwater availability threatens human health, agriculture, and industrial activities [1]. The rapid increase in population, climate change, and unsustainable water consumption have exacerbated this crisis, emphasizing the importance of finding efficient and sustainable water purification methods [2]. Seawater and brackish water desalination have emerged as viable solutions to meet the growing demand for clean water, offering a reliable alternative to conventional freshwater sources [3]. However, traditional desalination techniques, such as thermal distillation and osmosis technologies, face challenges related to high energy consumption and environmental constraints [4]. To address these limitations, researchers aspire to develop new membrane technologies that are more efficient and less harmful to the environment.

The demand for high-performance membrane-based processes had led to extensive research into advanced materials and fabrication techniques to enhance membrane functionality, selectivity, and durability. Several innovative approaches have been explored to improve membrane performance, including phase inversion, interfacial polymerization, and electrospinning [5]. Among these techniques, electrospinning has recently gained significant attention as an effective method for fabricating nanofibrous membranes with high and nearly uniform porosity, interconnected pore structures, and tunable surface properties.

Electrospinning is one of the recently developed processes for nanofiber production. It is considered a promising technology for the synthesis of optimum membrane structures for vital technological applications, including fuel cells, water electrochemical desalination, and water hydrolysis. In electrospinning technology, ultrafine nanofibers are formed out of a polymeric liquid solution under the influence of a high electric potential difference that can reach 30 kV. The induced electrostatic forces surpass the surface tension, forming a swirling conical jet (known as a Taylor cone) that swiftly advances towards the collector, thus resulting in minuscule fibres [6]. The process provides a very flexible technique, accommodating a spectrum of nanomaterial additives, such as metal oxides, carbon nanotubes, and polymers [7].

In electrospinning, there are several fundamental process parameters that affect the resulting nonwoven nanofiber structure. These process parameters include the process operating conditions, polymeric solution conditions, and some external environmental factors, such as ambient humidity levels. Proper control of these parameters is a necessity to produce fine fibres that are free from defects or polymer bead structures. A smoothly-formed Taylor cone is crucial for achieving a stable electrospinning process, as it regulates the morphology and properties of the resulting nanofibers [8].

The selection of materials for a certain membrane process is not a trivial decision. On the contrary, it is a challenging process because each application requires its own peculiar properties and characteristics. For water desalination and purification membrane processes, the selection of membrane materials becomes an essential factor that directly influences the efficiency and durability of the whole process. Notably, among the array of materials employed, polymeric materials emerge as prominent contenders. These include polysulfone (PSF), polycarbonate (PC), polyacrylonitrile (PAN), polyether sulfone (PES), polypropylene (PP), poly arylene ether nitrile ketone (PPENK), polyvinylidene fluoride (PVDF), polyimide (PI), and polytetrafluoroethylene (PTFE) [9].

Due to its outstanding properties including high hydrophobicity, chemical and thermal stability, and excellent mechanical properties, PVDF has assumed a unique position in many membrane-based processes. Recent developments in PVDF membrane applications in water and wastewater treatment, as well as other areas, such as membrane-based gas absorption and membrane distillation, have been the subject of several ongoing research activities [10]. For example, Li et al. [11] attempted to develop high-efficiency membranes for membrane distillation (MD) that are of high porosity, high hydrophobicity, and adequate mechanical strength for long-term operation. To enhance the mechanical properties and wetting resistance of PVDF membranes for MD of seawater, Kang-Jia et al. [12] have incorporated n-butylamine modified graphene oxide in PVDF flat-sheet and hollow fiber membranes. It was found that the additive led to PVDF flat-sheet membranes with better mechanical properties than those of the conventional PVDF–graphene oxide. Tonghu et al. [13] attempted a hybrid fabrication method for PVDF membranes by careful choice of a water-soluble diluent for the membrane to avoid membrane pore wetting and to ensure high salt rejection in MD applications.

This study represents the first part of an accomplished comprehensive research work on the development of optimum permselective membranes for water desalination using the capacitive electrochemical approach. Specifically, the study addresses the optimization, preparation, and characterization of PVDF-based mixed matrix membranes for effective use in electrochemical capacitive desalination. PVDF was selected due to its thermoplastic nature and piezoelectric effect. It can easily be dissolved in dimethylformamide (DMF), and acetone, then transformed into nanofibers via the electrospinning technique. PVDF nanofibrous membranes are successfully produced without defects and bead formation under typical electrospinning operational parameters. The permselectivity features of the produced membranes are established in the second part of the study, where these membranes are experimentally utilized in a capacitive electrochemical desalination study.

The hydrophobic behavior of PVDF could be advantageous for certain desalination applications, yet disadvantageous for others. For this reason, several additives can be blended with PVDF to modify its structure and enhance its hydrophilicity. The additives can be used to increase the conductivity, surface charge, and mechanical strength. Zinc oxide (ZnO) is a well-known semiconductor with a unique electronic structure and physical properties. It is mechanically robust and can strengthen the mechanical strength of the PVDF polymer. It can also control roughness and hydrophilicity of the membrane due to the presence of oxygen atoms and its high surface polarity. This results in high ion adsorption rates and high membrane desalination efficiency [14].

Activated carbon (AC) is commonly used in filtration and desalination facilities due to its high porosity and surface area. It increases the adsorption capacity of membranes by capturing a variety of contaminants in wastewater. In addition, it modifies the PVDF membrane to have a hydrophilic nature because of its surface functional groups, including hydroxyl (-OH), carbonyl (C=O), and carboxyl (-COOH) groups [15]. There are two types of carbon nanotubes, i.e., single-walled carbon nanotubes (SWCNTs), which contain one layer rolled into a tube, and multiwalled carbon nanotubes (MWCNTs), where multiple layers of graphene are curled into one cylinder. Usually, MWCNTs are more conductive due to their structure and interconnected bundles. The integration of MWCNTs with PVDF in this study is expected to promote better membrane performance as the conductivity and thermal stability increase significantly [16].

Graphen oxide (GO) is formed by the oxidation of graphite and can easily be dispersed in solvents, such as DMF and acetone. It is very effective when blended with PVDF as it improves the electrical conductivity, surface charge, and stability. GO also enhances the porosity and pore size distribution of the PVDF membrane to be effectively used in different desalination applications [17]. MOFs are porous materials made of metal clusters and organic ligands or linkers which are arranged in a three-dimensional structure. The most common MOF used for water desalination is zeolitic imidazolate framework-8 (ZIF-8). It is composed of zinc ions linked with 2-methylimidazole. ZIF-8 can effectively modify the structure of PVDF to achieve remarkable salt rejection, as it can block large ions and allow only sodium ions to pass through the membrane. It can also offer high porosity and a large surface area because of its well-arranged structure [18].

In this work, we provide recipes for fabricating membranes with various additives for possible use in capacitive electrochemical water desalination. Specifically, the study addresses the optimization, preparation, and characterization of PVDF polymeric membranes using the electrospinning technique. The synthesized membranes are characterized by a conductivity test, scanning electron microscopy (SEM), Fourier transform infrared spectroscopy (FTIR), energy-dispersive X-ray spectroscopy (EDX), porosity and pore size distribution, zeta potential, and contact angle tests. The conductivity test is considered crucial to establish the membrane’s ionic conductivity, whereas the SEM is considered essential to provide information about the membrane’s surface morphology [19]. FTIR provides information on the membrane’s matrix, including chemical compositions and functional groups, while EDX identifies the elemental structure of the membrane. Porosity and pore size distribution tests give an indication about the pores incorporated within the membrane. The zeta potential test is essential to determine the surface charge of the membrane, and the contact angle analysis is performed to identify the surface wettability and water uptake levels.

## 2. Experimental Section

### 2.1. Materials

The chemical materials used in this study were supplied by Sigma Aldrich, St. Louis, MO, USA. The reagents were used without any further purification. Table 1 presents some relevant properties for the chemical materials involved.

### 2.2. Preparation of PVDF Solution

The first step in membrane synthesis using the electrospinning technique is the preparation of a proper polymer solution that can be easily electrospun in the electrospinning machine. As Table 2 shows, six different PVDF-based membranes were synthesized. Each of these membranes were fabricated from a solution of the PVDF polymer and the solvents, as indicated in Table 2. The mixture was heated under continuous magnetic stirring at 70 °C for one hour. The solution was then allowed to cool down to room temperature (approximately 26 °C). The cold mixture was then stirred at room temperature for about 24 h. This experimental procedure was found to be necessary to ensure an easily spun fiber. The prepared PVDF solution was stored in a tight container to avoid contamination. In the case of additives, the same procedure was followed with the respective additive being included with the mixture before the heating step. For additives to be of value, they must produce effects when incorporated at low concentrations. Otherwise, they could become economically prohibitive, or they could produce major structural effects that might negate their use. Typically, high-cost additives are incorporated at concentrations below 1 wt.%. In addition to the no-additive case, additives in this study were investigated at two levels, that is, 0.18 wt.% and 0.45 wt.%.

### 2.3. Membrane Preparation by Electrospinning Technique

All the PVDF membranes with the different additives were prepared using the electrospinning device (Inovenso Ltd., Istanbul, Turky). The process involved forming a polymer solution, as indicated in Section 2.2, as a first step. This solution was then pumped into the electrospinning device under the influence of a high potential, which was 25 kV in this study, as shown in Table 2. Initially, 10–20 mL of the solution was introduced into a syringe specific to the electrospinning apparatus. The syringe was equipped at its tip with two nozzles, and the syringe assembly was connected to a high precision pump through a silicon rubber capillary tube. A flow rate in the range of 1–20 mL/h was used. The distance between the needle and collector drum (where the voltage difference is applied) was fixed at 15 cm. When the liquid reached the nozzle, it was attracted towards the drum under the influence of the high voltage, forming a (Taylor cone) jet stream impinging on the rotating collector drum, thus forming the required fibers. The rotating drum on which the membrane formed usually rotates within a range of 300–500 rpm and moves right and left horizontally to improve membrane homogeneity.

### 2.4. Membrane Characterization

All the prepared membranes were characterized using the following methods and testing procedures: electron scanning microscopy, electrical conductivity tests, field emission scanning electron microscopy (FESEM), Fourier transform infrared spectroscopy (FTIR), energy-dispersive X-ray spectroscopy (EDX), porosity and pore size distribution, zeta potential, and contact angle tests. The conductivity test was performed to study the effects of the involved additives on membrane’s ability for conducting ions. FESEM (FE-SEM, Model: TESCAN MAGNA UHR SEM, Oxford Instruments, Oxford, UK) results can provide information about the membrane’s surface morphology. FTIR (SHIMADZU IR Tracer 100, Kyoto, Japan) was used to investigate the membrane’s matrix, including chemical compositions and functional groups, while EDX and colour mapping (FE-SEM, Model: TESCAN MAGNA UHR SEM, Oxford Instruments UK) were utilized to identify the elemental structure of the membrane. Porosity and pore size distribution tests, which give an idea about the pores incorporated within the membrane, were measured using the wet agent method (Porous Materials, Inc. (PMI), New York, NY, USA). The zeta potential (Anton Paar SurPASS 3, Graz, Austria) was evaluated to determine the surface charge of the membrane, whereas the contact angle, which identifies the surface wettability and water uptake levels, was measured using contact angle apparatus (KRÜSS Drop Shape Analyzer, Hamburg, Germany).

The membrane’s electrical conductivity was calculated using the following formula [19]:

(1) $$\sigma =\frac{t}{RA_{C}}$$

where σ denotes the conductivity (mS/cm), t is the thickness of the polymeric membrane (cm), R is the measured bulk resistance (Ohm), and $A_{C}$ is the cross-sectional area of the membrane $\left(cm^{2}\right)$. The membrane’s porosity (ε) was calculated using the following Equation (2):

(2) $$\varepsilon =\frac{m_{w}-m_{d}}{\rho_{Galwick}\;*\;V_{d}}$$

where $m_{w}$ and $m_{d}$ are the weights of the wet and dry membranes, respectively, $\rho_{Galwick}$ is the density as measured according to the Galwick^TM^ test, and $V_{d}$ is the volume of the dry membrane.

## 3. Results and Discussion

### 3.1. Membrane Morphology Analysis

**Figure 1:**
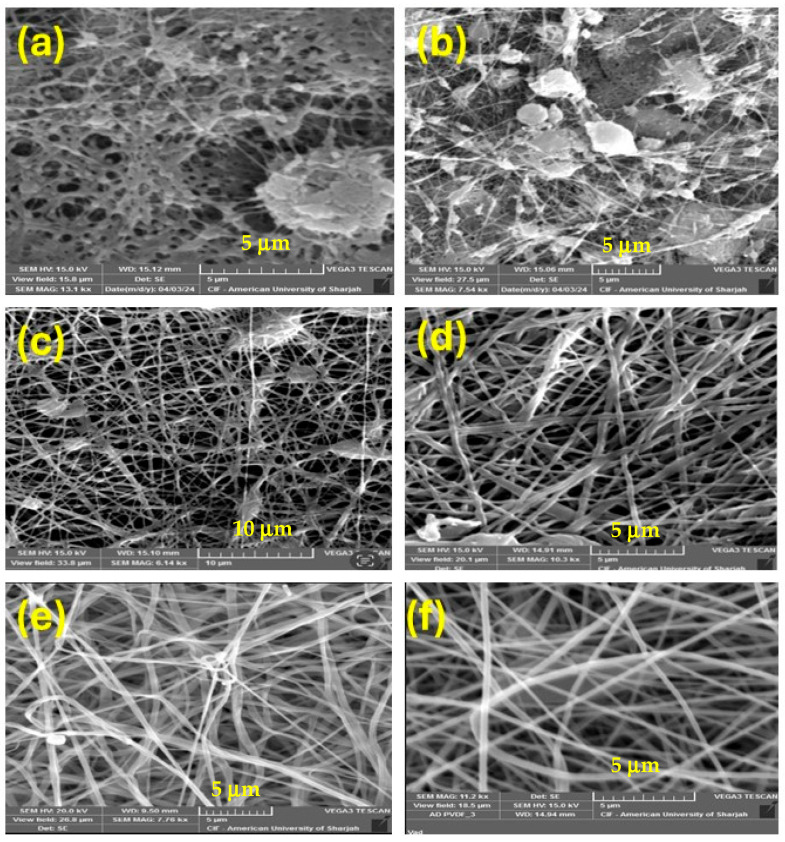
SEM images of the PVDF membranes fabricated in this study: (**a**) 20 mL/h, 9:1 DMF/AC, (**b**) 10 mL/h, 9:1 DMF/AC, (**c**) 5 mL/h 7:3 DMF/AC, (**d**) 3 mL/h, 7:3 DMF/AC (**e**) 2 mL/h, 7:3 DMF/AC, and (**f**) 5 mL/h, 6:4 DMF/AC.

The SEM images of the prepared membranes are shown in Figure 1a–f. The SEM images shown in this figure were prepared under similar conditions except for different flow rates and DMF/acetone ratios. These SEM images indicate some failed attempts at membrane fabrication, as revealed by the appearance of fused regions. From Figure 1c–e, the membranes fabricated with a solvent ratio of 7:3 and flow rates of 5, 2, and 1 mL/h show some variability. When the pump’s flow rate changes from 10 to 5, 2, and 1 mL/h, and the solvent ratio changes from 9:1 to 7:3, better fibers are formed, although some small beads are still observed. In Figure 1f, it is shown that when using a solvent ratio of 6:4 and a flowrate of 5 mL/h, uniform nanofibers were produced with no visible bead formation. This is considered the optimal condition for obtaining a uniform membrane structure. Thus, the optimal combination of experimental conditions is considered to be that corresponding to column (F) in Table 2. Therefore, in this work, all the membranes with their various additives were fabricated using the optimized conditions listed in column F of Table 2.

**Figure 2:**
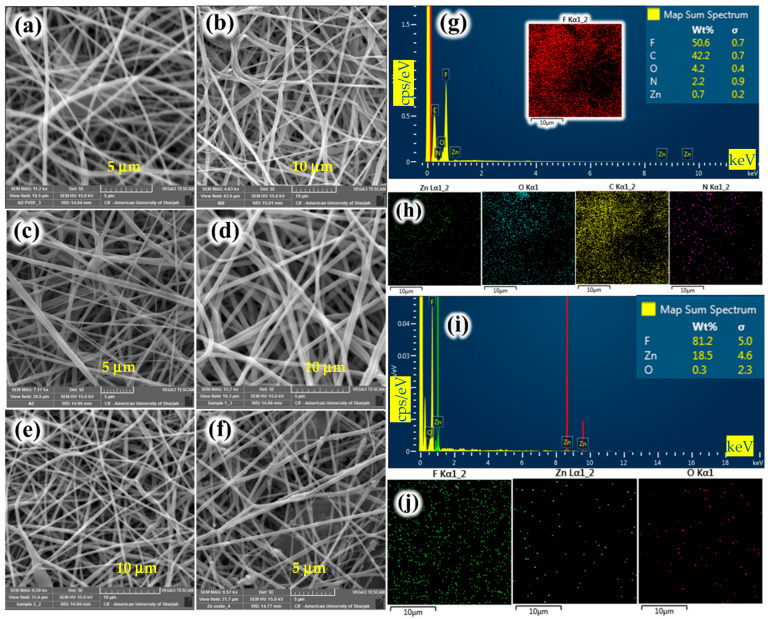
SEM images for PVDF membranes with different additives (base case conditions are those of case F in Table 2): (**a**) Pure PVDF, (**b**) PVDF/GO, (**c**) PVDF/AC, (**d**) PVDF/MOFs, (**e**) PVDF/CNTs, (**f**) PVDF/ZnO, and (**g**–**j**) EDX and color mapping images for PVDF/MOFs and PVDF/ZnO, respectively.

**Figure 3:**
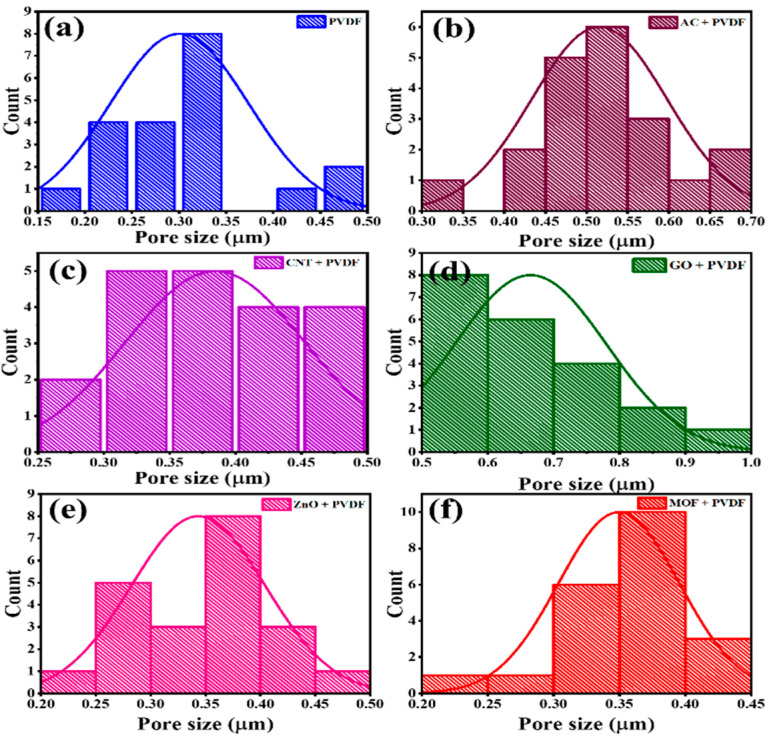
Pore size distribution for the prepared membranes: (**a**) PVDF membranes, (**b**) AC/PVDF, (**c**) CNTs/PVDF, (**d**) GO/PVDF, (**e**) ZnO/PVDF, and (**f**) MOFs/PVDF.

The SEM images portrayed in Figure 2a–f indicate a reasonable uniformity in the surface morphology. The EDX spectra, along with the dispersed elements and color mapping, are shown Figure 2g–j. The EDX and color mapping images in Figure 2g,h are representative of PVDF/MOF membranes. Similarly, Figure 2i,j show the EDX and color mapping images for PVDF/ZnO membranes. Apparently, the occurrence of both NPs is confirmed with a high purity. To estimate the average diameter of the incorporated pore size, an open-source software (ImageJ software, Version 1.54 [20]) was employed to calculate the average pore size based on their SEM images. For each fabricated membrane, pore size measurements were obtained by randomly selecting and analyzing 20 or 21 individual spots from high-resolution SEM micrographs. Image analyses conducted using the ImageJ software [20] were used to generate pore size distribution histograms, as illustrated in Figure 3a–f. As shown in this figure, the PVDF/GO membrane (Figure 3d) exhibits the largest mean pore size of approximately 2.086 μm, along with a relatively narrow distribution, suggesting a high degree of uniformity in the pore structure. The estimated pore size distribution results are presented in Table 3.

**Figure 4:**
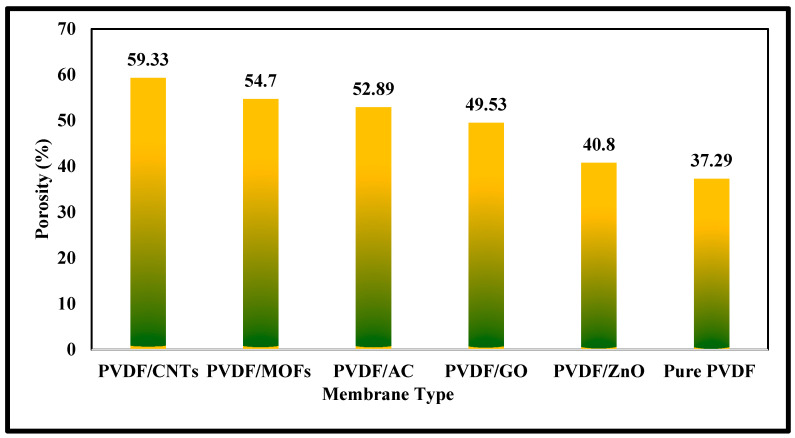
Porosity results for mixed matrix membrane with different additives.

The experimental results from the porosity measurements are represented in Figure 4. The PVDF/CNTs membrane is the most porous membrane, with a porosity of 59.33%. This can be partly attributed to the multiwalled carbon nanotube structure. The PVDF/MOFs membrane comes next with a porosity of 54.70%, which also illustrates a remarkable porosity due to its porous framework. The PVDF/AC membrane’s porosity is 52.89%, as it has a microporous network. Although PVDF/GO is the most conductive membrane, its porosity is slightly lower than others (49.53%). The PVDF/ZnO membrane has a dense crystalline structure with fewer internal pores (40.80%). These results emphasize the great impact of additives on the porosity of PVDF membranes.

### 3.2. Functional Groups-FTIR Analysis

**Figure 5:**
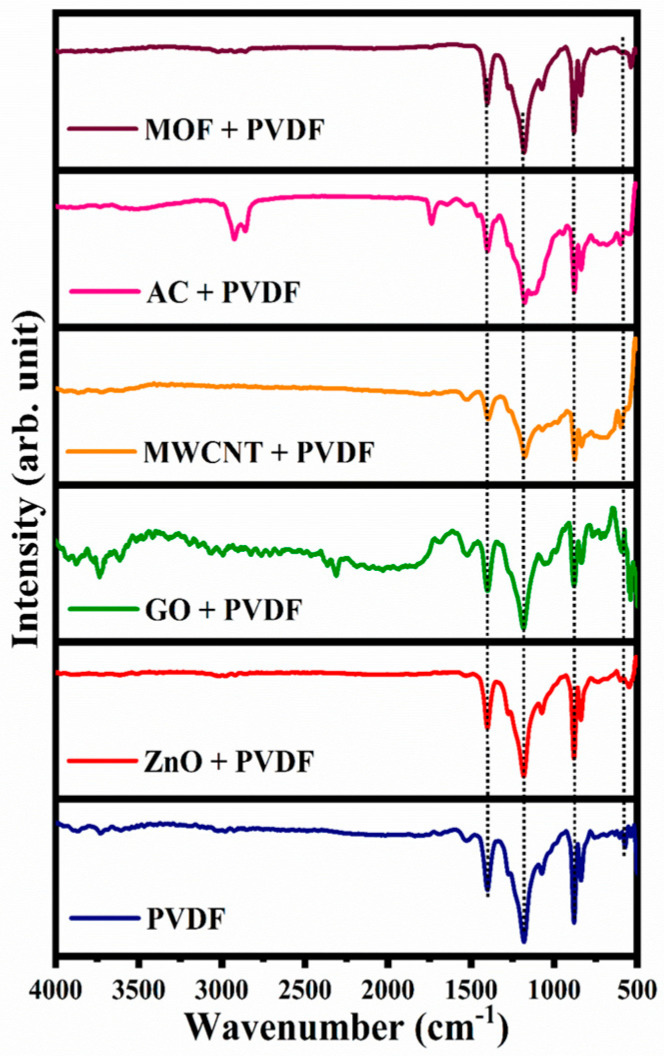
FTIR spectra for the different prepared membranes.

The identification of functional groups was determined by FTIR analysis, and the respective spectra are presented in Figure 5. From the results, the recorded spectra with desired peaks represent specific functional groups. These peaks emphasize the presence of the functional groups intended by the additives incorporated within the PVDF membrane’s structural skeleton. This analysis also provides insights into potential chemical interactions between the additives and the polymer matrix. The obtained shifts in peak positions or changes in intensity can be linked to modifications of the molecular structure [21]. This ensures that the additives effectively influence the membrane’s chemical and functional properties. The mixed matrix membrane was prepared with different additives, and Figure 5 showcases the FTIR spectra for these prepared membranes. The two common broad bands at 1431 cm^−1^ and 1172 cm^−1^ represent the C-H bond that is present in all membranes, explaining the existence of PVDF [22]. The FTIR spectra of the pure PVDF membrane revealed several key patterns. The stretching vibration of PVDF is observed at 1172 cm^−1^. The peaks at 878 and 840 cm^−1^ are related to the asymmetric vibration of C-C-C and C-F stretching vibrations, respectively [22]. For the ZnO/PVDF membrane, the peak at 549 cm^−1^ is probably attributed to the stretching vibration of the Zn-O bond [23]. For the spectra recorded for GO/PVDF membrane, the peaks at 2300 and 3757 cm^−1^ refer to the symmetric ring deformation of epoxy groups and C-O stretching vibrations of alkoxy groups [24]. In the case of the PVDF/CNTs membrane, the distinct adsorption peaks at 1224 cm^−1^ and 1381 cm^−1^ were attributed to the C=C and CO-H groups, respectively. A new band is observed at 1418 cm^−1^ that is relevant to the presence of the -OH and -COOH groups on the oxidized MWCNT’s surface [25]. For the PVDF/AC membrane, a broad peak was observed at 2746 cm^−1^, associated with O-H stretching vibrations because of water molecules. The peak at 1622 cm^−1^ was assigned to C=O stretching vibrations from carboxylic acids [26]. An overlapped band at 974 cm^−1^ was associated with C-H vibrations [27]. For ZIF-8 peaks in the PVDF/MOFs spectrum, the peak at 651 cm^−1^ correlated with the C-N bond vibration in the imidazole ring [28]. The band at 420 cm^−1^ is also observed [29]. From these results, it is safely concluded that the spectra of the synthesized PVDF membranes with the additives confirmed the occurrence of the intended functional groups in the prepared membranes.

### 3.3. Membrane Electrical Properties

**Figure 6:**
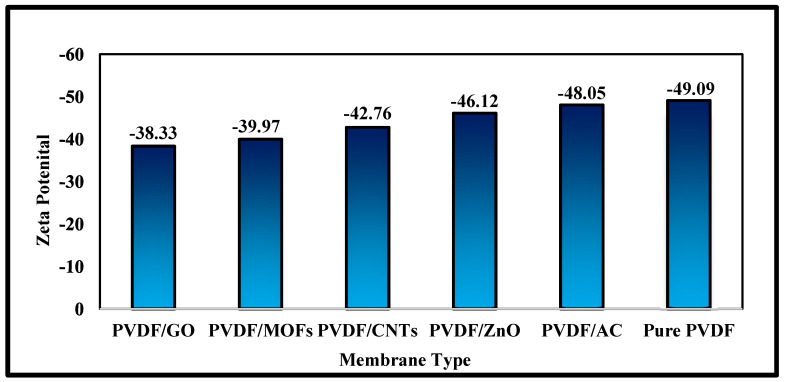
Zeta potential measurements for the different synthesized membranes.

The surface charges of the prepared membranes with the additives were evaluated by zeta potential analysis. The zeta potential analysis (Figure 6) showed negative values, which correlated with the presence of sulfonic groups on the membranes surface, confirming that sulfonation was effectively achieved. The membranes were ordered from the lowest to the highest absolute zeta potentials as follows: PVDF/GO (−38.33), PVDF/MOFs (−39.97), PVDF/CNTs (−42.76), PVDF/ZnO (−46.12), PVDF/AC (−48.05), and pure PVDF (−49.09). Thus, upon the addition of functional materials, the absolute values of zeta potential decrease, indicating alterations in the surface charge characteristics or some surface charge neutralization. The least absolute zeta potential for the PVDF/GO membrane (−38.33 mV) is probably attributed to the presence of oxygen-containing functional groups (such as carboxyl, hydroxyl, and epoxy) on the GO nanosheets. Although these groups are ionizable and typically expected to enhance the negative charge, the net reduction in surface negativity compared to pure PVDF may result from the masking of PVDF’s native surface groups and even the possible aggregation effects of GO. For PVDF/MOFs (−39.97 mV), the decrease in negativity suggests the influence of metal ions (e.g., Zn^2+^ from ZIF-8) which may partially neutralize surface charge or introduce weakly positively charged sites. The coordination between metal ions and the polymer matrix may also hinder full ionization of acidic groups. The PVDF/CNTs membrane exhibits a slightly more negative zeta potential (−42.76 mV), which may be attributed to acidic oxygen functionalities, such as carbonyl or hydroxyl groups, that could result from the potential surface oxidation of CNTs during processing. A more pronounced increase in the membrane negativity is observed for the PVDF/ZnO (−46.12 mV); ZnO nanoparticles may incentivize hydroxylation of the membrane surface, leading to an increased negativity. Similarly, PVDF/AC (−48.05 mV) maintains a zeta potential close to that of pure PVDF, which can be explained by the high surface area and abundance of acidic surface functional groups (e.g., carboxylic and phenolic) in activated carbon. These groups are likely to dissociate in aqueous conditions, reinforcing the membrane’s negative surface potential.

**Figure 7:**
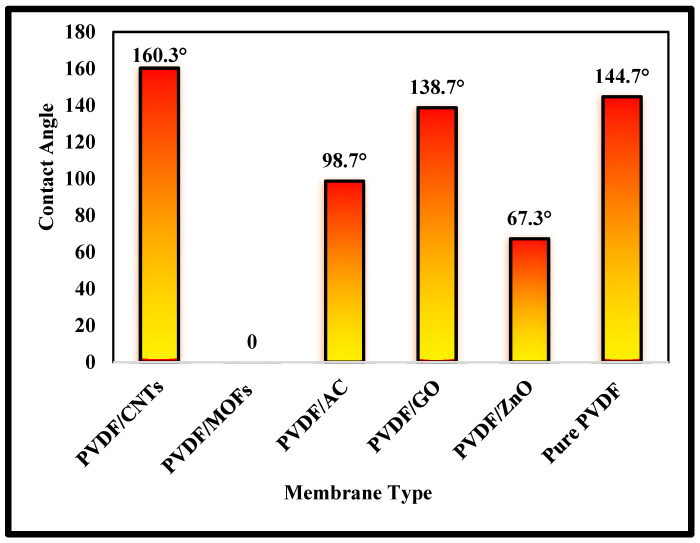
Contact angle for the different synthesized membranes.

The contact angle measurements were conducted to determine the wettability of the prepared membranes and to establish the degree of hydrophobicity/hydrophilicity of the prepared membranes (Figure 7). The addition of additives into the hydrophobic PVDF polymeric membrane, which has a contact angle of 144.7°, showed variations in the resulting contact angle. The contact angle test of the PVDF/CNTs membrane showed a contact angle of 160.3°, indicating a high degree of hydrophobicity gained by incorporating the CNTs into the polymer matrix. However, the PVDF/GO contact angle was found to be 138.7°, which is less than that of the raw PVDF membrane, indicating a reduction in hydrophobicity. The contact angle of PVDF/AC was measured at 98.7°, while the contact angle of PVDF/ZnO was 67.3°. The PVDF/MOFs did not show any measurable contact angle, as the droplet immediately deformed before it could be measured.

The conductivities of the mixed matrix membranes were tested to identify the most conductive formulation. The conductivity test was performed using a suitable potentiostat. The results presented in Table 4 show the conductivity values for each membrane at two levels of additive composition, that is, 0.18 wt.% and 0.45 wt.%. It is obvious that all the membranes gained higher conductivity at the higher level of additive concentration. As the table shows, the PVDF/GO membrane at an additive composition of 0.45 wt.% exhibits the highest conductivity (5.6106 mS/cm), making it the most conductive membrane among the ones investigated in this study. This is probably because of its excellent electron mobility and dispersion. It is worth mentioning that these measurements were conducted at a room temperature of about 26 °C. These findings underline the importance of both the choice of additives and their concentration in optimizing the performance of PVDF membranes. Furthermore, the enhanced conductivity observed with higher concentrations is key to achieving the desired electrical and mechanical properties for specific applications, such as saline water desalination. The correlation between conductivity and additives provides valuable insights for future membrane design, where a balance between conductivity, stability, and other functional properties must be maintained. Table 4 also shows that CNTs and AC enhance conductivity significantly, especially at higher loadings of the additive. MOFs and ZnO, however, provide moderate improvements, probably due to lower intrinsic conductivity and weaker network formation in the polymer matrix. It is also observed that membrane thickness increases with additive loading, but conductivity gains typically outweigh the thickness penalty. The increase in conductivity with thickness in the electrospun mixed matrix membranes is primarily due to improved conductive network formation and interconnectivity of nanofillers, which outweigh the classical geometric effects observed in bulk conductive materials.

## 4. Conclusions

PVDF-based mixed matrix membranes incorporated with several additives were successfully prepared by electrospinning techniques. The optimum conditions for fabricating suitable membranes with uniform fiber appearance were experimentally established. All the prepared membranes were characterized by various spectroscopic techniques. The presence and effects of various functional groups were confirmed. The FESEM analysis revealed that the synthesized membranes showed uniform surface morphology with well dispersed additives on the fibers. The elemental composition and the corresponding color mapping images indicated that the respective elements of the additives were successfully integrated into the membrane structure. The PVDF/GO membrane exhibits the largest mean pore size of approximately 2.086 μm, along with a relatively narrow distribution, suggesting a high degree of uniformity in the pore structure. The highest porosity found was 59.33% for the PVDF/CNTs membrane. Based on the findings of this study, most of the PVDF-based mixed matrix membranes studied can serve as acceptable membranes for employment in electrochemical capacitive desalination. In terms of characteristics, PVDF integrated with GO could be the best among all of the membrane formulations investigated in this study.

## Figures and Tables

**Table 1:** Properties of chemical materials used in this study.

Material	Properties
Zinc oxide particles (ZnOs)	<5 μm particle size, 99.9% purity
Graphene oxide particles (GOs)	0.5–10 μm size, 0.55–50 nm thickness, industrial grade, better than 97% purity
Multiwalled carbon nanotubes (MWCNs)	50–90 nm diameter, better than 95% purity (carbon basis)
Zeolitic imidazolate framework-8 (ZIF-8)	Zinc ions linked with 2-methylimidazole
Activated carbons (AC)	Research grade purity
Dimethylformamide (DMF)	Better than 99.9% purity
Acetone	Better than 99.9% purity
Sulfuric acid (H_2_SO_4_)	Better than 98% purity

**Table 2:** Electrospinning parameters used in the synthesis of PVDF membranes.

Parameters	PVDF Samples					
	A	B	C	D	E	F
Mass of polymer (g)	1	1	1	1	1	1
DMF/acetone volume ratio (mL/mL)	9:1	9:1	7:3	7:3	7:3	6:4
Polymer concentration (wt.%)	10	10	10	10	10	10
Needle–collector distance (cm)	15	15	15	15	15	15
Voltage (kV)	25	25	25	25	25	25
Drum speed (rpm)	300	300	300	300	300	300
Syringe capacity (mL)	10	10	10	10	10	10
Flow rate (mL/h)	20	10	5	3	2	5
Duration of electrospinning (h)	0:30	1:00	2:00	5:00	10:00	2:00

**Table 3:** Average pore size and average fiber diameter as calculated by ImageJ open-source software [20].

Membrane	$\mathrm{Average}\;\mathrm{Pore}\;\mathrm{Size}\;(\mu_{m})$	$\mathrm{Average}\;\mathrm{Fiber}\;\mathrm{Diameter}\;(\mu_{m})$
Pure PVDF	0.298	0.931
PVDF/AC	0.515	1.617
PVDF/CNTs	0.384	1.207
PVDF/GO	0.664	2.086
PVDF/MOFs	0.349	1.096
PVDF/ZnO	0.343	1.077

**Table 4:** Measured conductivities of different PVDF-based membranes (except for the composition level, the experimental conditions and parameters are those corresponding to case F in Table 2).

Mixed Matrix Membrane	Composition (wt.%)	Resistance (Ohm)	Thickness (cm)	Conductivity (mS/cm)
PVDF/no additives	-	195.6122	0.0457	0.3677
PVDF/GO	0.18	56.1237	0.0533	1.4995
PVDF/GO	0.45	23.3349	0.0832	5.6106
PVDF/AC	0.18	84.8904	0.0802	1.4983
PVDF/AC	0.45	59.3602	0.0891	1.7742
PVDF/ZnO	0.18	85.4439	0.0511	0.9414
PVDF/ZnO	0.45	67.0813	0.0821	1.9220
PVDF/MOFs	0.18	109.9002	0.0612	0.8761
PVDF/MOFs	0.45	88.9310	0.0753	1.3349
PVDF/CNTs	0.18	112.0672	0.0834	1.7311
PVDF/CNTs	0.45	55.8628	0.0923	2.6033

## Data Availability

Data are contained within the article.
